# Radiation oncology at crossroads: Rise of AI and managing the unexpected

**DOI:** 10.1002/acm2.70043

**Published:** 2025-02-17

**Authors:** Mohammad Bakhtiari

**Affiliations:** ^1^ Department of Radiation Oncology WellSpan Health Chambersburg Pennsylvania USA

**Keywords:** artificial intelligence, cognitive diversity, high reliability organization, patient safety, psychological safety, radiation oncology, real‐time adaptability

## Abstract

Integrating artificial intelligence (AI) into radiation oncology has revolutionized clinical workflows, enhancing efficiency, safety, and quality. However, this transformation comes with a price of increased complexity and the emergence of unpredictable events. This letter proposes a framework based on high reliability organization (HRO) principles for managing real‐time, unforeseen events. The framework emphasizes proactive risk assessment, adaptive teamwork at the situation assessment point, and reactive learning through incident analysis by placing human‐centered decision‐making at the core. Integrating cognitive diversity, psychological safety, and emotional intelligence fosters collective intelligence, enabling teams to navigate AI‐driven complexities while safeguarding patient safety.

## 1 INTRODUCTION

Artificial intelligence (AI) in healthcare has been extensively studied, addressing risks such as bias, lack of transparency, and integration challenges. AI applications in radiation therapy heavily depend on clinical training data, which introduces variability and systematic errors that are challenging to detect. For instance, auto‐segmentation errors may arise from anatomical variations or imaging inconsistencies. As AI expands beyond auto‐segmentation to areas like dose prediction, treatment delivery, and quality assurance, it is expected to bring unforeseen complexities.^1^ Recent guidelines provide structured pathways for AI implementation in clinical workflows, emphasizing validation, robustness, and clinical integration.^2^ Additionally, quality assurance frameworks for AI applications in radiation therapy focus on lifecycle management and validation before clinical use.^1^

While these approaches guide AI development and deployment, they address upstream processes and system‐level governance. In contrast, this letter focuses on the clinical application of AI in radiation oncology, where human supervision and real‐time frontline interventions are critical. Integrating AI into workflows introduces new complexities, requiring healthcare teams to manage critical moments when unexpected events occur, demanding timely resolution. Following high reliability organization (HRO) principles,^3^ this framework emphasizes human‐centered adaptability through psychological safety (the freedom to voice concerns),^4^ cognitive diversity (leveraging varied perspectives),^4^ emotional intelligence (managing emotions to enhance collaboration),^5^ and collective intelligence (synergistic team problem‐solving),^6^ enabling teams to complement AI systems and respond effectively in high‐stakes scenarios.

Complexity, an inherent property of adapting to dynamic environments, is both a strength and a challenge in radiation oncology. While improving safety, quality, and efficiency, it also increases the potential for unwanted events. Acknowledging complexity involves accepting the system's uncertain nature. Current safety strategies, including TG 100,^7^ incident learning systems (ILS), and root cause analysis (RCA),^8^ primarily address predictable risks and past events. However, the ability to manage the unexpected in an inherently unpredictable system is still a critical gap as AI becomes integral to clinical workflows.

The aviation industry offers powerful lessons in managing the unexpected:

1. **Adaptability**: The “Miracle on the Hudson” (US Airways Flight 1549) exemplifies adaptability and psychological safety. When both engines failed, the crew landed the plane on the Hudson River, proving technical skill and an environment that empowered decisive action under pressure.^9^
2. **The danger of over‐adherence to rigid rules**: The crash of American Airlines Flight 587 in 2001 highlights how rigidly applying training techniques can escalate manageable events into disasters. Shortly after takeoff, the first officer's aggressive use of the rudder, following specific training, led to the vertical stabilizer breaking off in response to wake turbulence. This highlights the importance of contextual judgment in responding to unexpected events.^10^
3. **The danger of over‐reliance on automation**: The crash of Air France Flight 447 in 2009 illustrates the risks of automation dependency. When the autopilot disengaged, the crew's lack of situational awareness and over‐reliance on automated systems contributed to the tragedy.^11^

In radiation oncology, these lessons emphasize the critical role of psychological safety, cognitive diversity, emotional intelligence, and collective intelligence in addressing unforeseen challenges AI technologies pose.

## 2 THE FRAMEWORK

**FIGURE 1:**
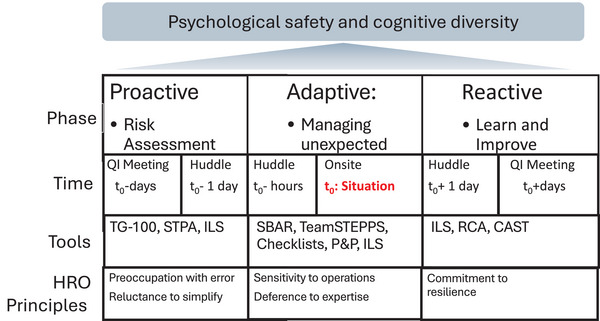
Framework for managing the unexpected in radiation oncology: This framework outlines the proactive, adaptive, and reactive (retrospective) phases of managing AI‐driven complexities, emphasizing the interplay of preventive measures, situational assessments, adaptability, and human‐centered decision‐making. AI, artificial intelligence; CAST, causal analysis based on systems theory; ILS, incident learning systems; P&P, policy and procedures; QI, quality improvement; RCA, root cause analysis; SBAR, situation‐background‐assessment‐recommendation; STPA, system‐theoretic process analysis; TeamSTEPPS, team strategies and tools to enhance performance and patient safety.

A situation assessment point is a critical juncture in radiation oncology where complex and unpredictable issues require careful evaluation and timely resolution. While the nature of these challenges cannot always be anticipated, examples include addressing gaps in critical structures identified during imaging or resolving procedural oversights, such as missing signatures on updated prescriptions, to ensure patient safety and treatment accuracy. Rooted in HRO principles,^3^ this framework addresses **proactive, adaptive,** and **reactive** phases to ensure a structured yet flexible approach to managing the unexpected [Figure 1].

### 2.1 Proactive phase (days before situation assessment point)

Key actions to mitigate risks include:

- Conducting robust risk assessments, such as TG 100^7^ or system‐theoretic process analysis (STPA).^12^
- Establishing preventive measures, including checklists and QA programs.^8^
- Ensuring readiness through peer reviews, contour rounds, and safety checks.
- **Huddle the day before**: Confirm safety requirements, address unresolved issues, and complete peer reviews and QA checks for the next day's cases.^13^

### 2.2 Adaptive phase (same day of situation assessment point)

This phase bridges pre‐planned strategies and real‐time situations with critical situational assessments:

- **Pre‐treatment same‐day huddle (hours before)**: Conduct a final review of safety‐critical tasks, including equipment calibration, procedural checks, and cross‐verifying AI predictions against baselines.
- **Situation assessment point, at the center of human ingenuity**: At the situation assessment point, preventive measures provide foundational safeguards:
  - **Preventive measures**: Force functions, checklists, and protocols serve as baseline safety systems.
  - **Team adaptability**: The team's collective intelligence enables dynamic responses to unanticipated challenges. Tools such as SBAR (Situation‐Background‐Assessment‐Recommendation)^14^ and TeamSTEPPS (Team Strategies and Tools to Enhance Performance and Patient Safety)^15^ under collective intelligence can guide the team through high‐pressure situations. The Physicist of the Day (POD) is essential in leading the team through the unexpected by assessing the situation and creating a safe environment for leveraging the team's inputs. Effective handoffs, clear communication, and collaborative problem‐solving frameworks enhance team adaptability and ensure timely and effective responses.

For example, if during the patient setup imaging, the team notices a gap between the brainstem and spinal cord (generated by automated AI auto‐segmentation), the team pauses the treatment, assesses the situation, evaluates the impact of the missing contours, and determines the next steps to take. Similarly, addressing procedural oversights, such as a missing signature from a last‐minute prescription change, requires careful evaluation to prevent unintended dosing.

### 2.3 Reactive phase (day(s) after)

Post‐event analysis fosters continuous learning:

- **Debriefings at the next day huddle**: Capture insights from the previous day's successes, near misses, and incidents.^13^, ^16^
- **RCA** and **ILS at a later time**: Inform systematic improvement by finding gaps and reinforcing best practices.

Systematic improvements are incorporated into preventive measures, completing one of the infinite Plan‐Do‐Study‐Act (PDSA) cycles.

## 3 CONCLUSION

As AI continues to shape radiation oncology, existing guidelines and quality assurance frameworks provide essential foundations for its deployment.^1^, ^2^ This letter extends these efforts by focusing on real‐time situation assessment. By fostering psychological safety, cognitive diversity, and emotional intelligence, the proposed framework emphasizes the role of collective intelligence in navigating AI‐driven complexities and safeguarding patient outcomes.

## AUTHOR CONTRIBUTIONS

Mohammad Bakhtiari: Sole investigator and author. Managed data, developed methodologies, conducted analysis, wrote and reviewed the manuscript.

## CONFLICT OF INTEREST STATEMENT

The author declares no conflicts of interest.
